# Use of a diagnostic Puumala virus real-time RT-PCR in an orthohantavirus endemic region in the Netherlands

**DOI:** 10.1128/spectrum.03813-23

**Published:** 2024-06-10

**Authors:** Felix Geeraedts, Mariska Wevers, Froukje Bosma, Maria de Boer, J. N. Brinkman, Corine Delsing, Corine GeurtsvanKessel, Barry Rockx, Adri van der Zanden, Gozewijn D. Laverman

**Affiliations:** 1 Laboratory for Medical Microbiology and Public Health, Hengelo, Overijssel, the Netherlands; 2 Department of Internal Medicine, Medisch Spectrum Twente, Enschede, Overijssel, the Netherlands; 3 Viroscience, Erasmus University Medical Center, Rotterdam, Zuid-Holland, the Netherlands; 4 Center for Infectious Disease Control, National Institute for Public Health and the Environment (RIVM), Bilthoven, Utrecht, the Netherlands; 5 Department of Internal Medicine, Ziekenhuis Groep Twente, Almelo/Hengelo, Overijssel, the Netherlands; London Health Sciences Centre, London, Ontario, Canada

**Keywords:** hantavirus, Puumala virus, serology, molecular methods, nucleic acid amplification test, zoonotic infections, nephrology, diagnostics

## Abstract

Laboratory diagnosis of orthohantavirus infection is primarily based on serology. However, for a confirmed serological diagnosis, evaluation of a follow-up serum sample is essential, which is time consuming and causes delay. Real-time reverse transcription polymerase chain reaction (RT-PCR) tests, if positive, provide an immediate and definitive diagnosis, and accurately identify the causative agent, where the discriminative nature of serology is suboptimal. We re-evaluated sera from orthohantavirus-suspected clinical cases in the Dutch regions of *Twente* and *Achterhoek* from July 2014 to April 2016 for the presence of Puumala orthohantavirus (PUUV), Tula orthohantavirus (TULV), and Seoul orthohantavirus (SEOV) RNA. PUUV RNA was detected in 11% of the total number (*n* = 85) of sera tested, in 50% of sera positive for anti-PUUV/TULV IgM (*n* = 16), and in 1.4% of sera negative or indeterminate for anti-PUUV/TULV IgM (*n* = 69). No evidence was found for the presence of TULV or SEOV viral RNA. Based on these findings, we propose two algorithms to implement real-time RT-PCR testing in routine orthohantavirus diagnostics, which optimally provide clinicians with early confirmed diagnoses and could prevent possible further invasive testing and treatment.

**IMPORTANCE:**

The addition of a real-time reverse transcription polymerase chain reaction test to routine orthohantavirus diagnostics may better aid clinical decision making than the use of standard serology tests alone. Awareness by clinicians and clinical microbiologists of this advantage may ultimately lead to a reduction in over-hospitalization and unnecessary invasive diagnostic procedures.

## INTRODUCTION

The genus orthohantavirus is a group of negative-sense single-stranded RNA viruses in the family of Hantaviridae, order of Bunyavirales, which also includes other hemorrhagic fever viruses such as the highly pathogenic Lassa and Crimean–Congo hemorrhagic fever viruses (1). Rodents are the natural reservoir of most orthohantaviruses, and transmission to humans generally occurs by inhalation of dried animal excreta. Orthohantavirus infections occur worldwide, and the presentation and severity depends on the type of orthohantavirus involved; orthohantaviruses in the Americas, including Sin Nombre orthohantavirus and Andes orthohantavirus, may cause a hantavirus pulmonary syndrome with case-fatality rates of approximately 40% (2); Hantaan orthohantavirus in Asia, Puumala, and Dobrava orthohantavirus in Europe, and Seoul orthohantavirus worldwide, may cause a hemorrhagic fever renal syndrome (HFRS) in varying degrees of severity with estimated mortality rates of 15%, <0.1%, 9%–12%, and <1%, respectively (3).

In the Netherlands, orthohantavirus infections in humans are predominantly caused by Puumala orthohantavirus (PUUV), although diagnosis is often based on routine serology assays, which do not differentiate between PUUV and Tula orthohantavirus (TULV). Historically, the highest incidence of orthohantavirus infections in the Netherlands is found in the *Twente* region, in the eastern part of the country, which coincides with the geographic presence of PUUV in its host, the bank vole (4, 5). Other orthohantaviruses prevalent in the Netherlands are TULV and Seoul orthohantavirus (SEOV). TULV is found in the common vole in the northern and southern areas of the country, but also in the *Twente* region (6, 7). Only few human cases of TULV infection have been described in the literature, and it is currently unknown whether TULV has caused infections in humans in the Netherlands due to cross-reactivity between TULV and PUUV in routine orthohantavirus serology (7). SEOV was found in wild brown rats (captured in 2013) in the adjacent region of *Achterhoek* in the province of Gelderland, on one occasion (8–10). Few human cases of Seoul virus infection in the Netherlands have been reported up to the present, which were associated with captivated rats (11–13).

PUUV infection can cause a milder form of HFRS called nephropatia epidemica (NE), characterized by acute kidney failure that usually resolves spontaneously within days. Whereas the clinical condition is usually relatively mild, the concomitant renal failure can be severe. In these circumstances, it is important to discriminate PUUV infection from other causes of acute kidney failure, particularly forms of rapidly progressive glomerulonephritis, which require invasive diagnostics and/or therapeutic procedures (kidney biopsy, hemodialysis, and/or plasmapheresis). Therefore, because of its good renal prognosis , the early confirmation of an orthohantavirus infection can prevent a kidney biopsy and/or the initiation of dialysis (14). Currently, serology is the mainstay of the detection of orthohantavirus infection. However, a positive anti-PUUV IgM and/or IgG in a single serum sample is not sufficient to prove an acute infection. Also, a negative serology result in the early phase of disease does not rule out an acute infection. In both cases, a follow-up serum sample after 2 weeks is needed to confirm the diagnosis, in case of an acute infection, by detecting a seroconversion or a significant rise in IgG titer. However, in clinical practice, investigation of a second serum sample is often not performed due to its limited clinical value once the acute phase has passed. Furthermore, orthohantavirus seroprevalence in the *Twente* region is estimated at 3.2%, which is significantly higher than the rest of the Netherlands (1.7%) (5), increasing the chance of incorrect interpretation of a positive IgG in a single serum sample as a sign of ongoing infection. Detection of viral RNA in the acute serum sample would confirm an orthohantavirus infection early in the clinical course. However, as the virus is present in the blood only shortly after development of symptoms, real-time reverse transcription polymerase chain reaction (RT-PCR) and other nucleic acid amplification tests (NAAT) are generally not used routinely.

Another limitation of serologic testing is that it does not discriminate between infection with PUUV and TULV infection due to cross-reactivity. Therefore, TULV infections may have been missed in the past. In addition, clinical cases of SEOV infection may have been overlooked in the past because routine serology testing for SEOV in the Netherlands started only after the reported endemicity in 2015, while evidence for SEOV presence in wild rats dates from 2013 (15), and serological data in wild rats captured in 2011–2012 may hint at the presence of SEOV in the Netherlands even earlier (9).

To evaluate the applicability of real-time RT-PCR in a routine orthohantavirus diagnostic work flow, sera from orthohantavirus-suspected patients in the region of the *Twente* and the northern part of the *Achterhoek* region were analyzed in retrospect for the presence of PUUV, TULV, and SEOV RNA.

## MATERIALS AND METHODS

### Serum samples

Serum samples from patients, in whom an orthohantavirus infection was suspected (*n* = 113) in the period 23 July 2014 to 1 June 2016, were evaluated in this study. We used serum samples that had been sent to the Laboratory for Medical Microbiology and Public Health for orthohantavirus serology in this period. Upon arrival in the laboratory, serum of the individual patient was aliquoted, and one sample was directly sent to the reference laboratory for serology, while the remainder was cryopreserved at −80°C, and later on used in the real-time RT-PCR test. Sera with positive and indeterminate serology results, as well as sera for which a follow-up sample was sent to the laboratory, are listed in Table 1. The day of onset of symptoms was derived from the clinical information stated by the clinician on the diagnostic order forms or by personal communication with the clinician.

**TABLE 1:** Characteristics of sera that tested positive or indeterminate for anti-Puumala orthohantavirus IgM and/or IgG, and sera with a second serum sample tested^*a*^

	First serum sample					Interval (days)	Second serum sample				Confirmation by serology
	IgG		IgM		Real-time RT-PCR		IgG		IgM		
**Serum**	**EIA**	**IF**	**EIA**	**IF**			**EIA**	**IF**	**EIA**	**IF**	
**1**	**P**	**-**	**P**	**-**	**P**		**-**	**-**	**-**	**-**	
**2**	**P**	**-**	**P**	**-**	**P**		**-**	**-**	**-**	**-**	
**3**	**P**	**-**	**P**	**-**	**P**		**-**	**-**	**-**	**-**	
4	P	-	P	-	N		-	-	-	-	
5	P	-	P	-	N		-	-	-	-	
6	P	-	P	-	N		-	-	-	-	
7	P	-	P	-	N		-	-	-	-	
8	P	-	P	-	-		-	-	-	-	
9	P	-	P	-	-		-	-	-	-	
10	P	-	P	-	-		-	-	-	-	
11	P	-	P	-	-		-	-	-	-	
12	N	-	P	-	N	49	N	-	N	-	N
13	N	-	P	-	N		-	-	-	-	
14	N	-	P	-	-		-	-	-	-	
15	N	-	P	-	-		-	-	-	-	
**16**	**-**	**P**	**-**	**P**	**P**	**12**	**-**	**P**	**-**	**P**	**P^#^**
**17**	**-**	**P**	**-**	**P**	**P**	**8**	**-**	**P**	**-**	**P**	**P**
**18**	**-**	**P**	**-**	**P**	**P**		**-**	**-**	**-**	**-**	
**19**	**-**	**P**	**-**	**P**	**P**		**-**	**-**	**-**	**-**	
**20**	**-**	**P**	**-**	**P**	**P**		**-**	**-**	**-**	**-**	
21	-	P	-	P	N		-	-	-	-	
22	-	P	-	P	N		-	-	-	-	
23	-	P	-	P	-		-	-	-	-	
24	-	P	-	P	-		-	-	-	-	
**25**	**N**	**-**	**Indet**	**-**	**P**	**11**	**P**	**-**	**P**	**-**	**P**
26	N	-	Indet	-	N	10	N	-	Indet	-	N
**27**	**P**	**-**	**Indet**	**-**	**-**	**49**	**P**	**-**	**N**	**-**	***P* ^##^**
28	P	-	N	-	N		-	-	-	-	
29	N	-	Indet	-	N		-	-	-	-	
30	N	-	Indet	-	N	18	N	-	N	-	N
31	N	-	Indet	-	N		-	-	-	-	
32	P	-	N	-	N		-	-	-	-	
33	N	-	N	-	N	21	N	-	N	-	N
34	N	-	N	-	N	7	N	-	N	-	N
**35**	**-**	**P**	**-**	**N**	**N**	**12**	**-**	**P**	**-**	**P**	**P**

^*a*^ Confirmed positive cases of acute infection with Puumala orthohantavirus, either by detection of viral RNA or confirmed by serology, are expressed in bold. Confirmation by serology was positive if there was a significant increase in IgG and/or IgM in the second serum sample {a fourfold rise in titer [immune fluorescence (IF)]}, or seroconversion of IgG and/or IgM [enzyme immunoassay (EIA)]. #The increase in IgG titer was twofold or more (titer 4,000 to ≥8,000), and no further titration was performed, but in combination with the clinical picture, a recent infection was concluded at the time. ##No seroconversion but sufficient increase in IgG ratio (4.71 to 5.21) combined with a decrease in IgM ratio (1.18 to 0.95) to conclude a recent infection. Shaded in gray: sera negative or indeterminate for IgM in the first sample. P, positive; N, negative; Indet, indeterminate; -, not done.

Sufficient amounts of cryopreserved serum samples to be re-evaluated with real-time RT-PCR for the presence of orthohantavirus RNA were available in 85 of the 113 suspected orthohantavirus cases. These serum samples were thawed, and viral RNA was extracted using the EasyMAGsystem of Biomerieux, according to the manufacturer’s instructions.

### Serology

The sera described above have initially been tested for antibodies reactive to PUUV IgG and IgM in the period 23 July 2014 to 1 June 2016 at the Department of Virology, Erasmus MC, Rotterdam, the Netherlands, using commercially available enzyme-linked immunosorbent assay (ELISA) (Hantavirus Puumala IgG/IgM ELISA, PROGEN Biotechnik, Heidelberg, Germany) or immune fluorescence (IF) (Anti-hantavirus IIFT, Euroimmun, Luebeck, Germany), according to the manufacturer’s instructions.

### Real-time RT-PCR

Detection of PUUV, TULA, and SEOV RNA by real-time RT-PCR was performed using primers and probes as described by Kramski et al. (16). In brief, real-time RT-PCR reactions were performed as a monoplex real-time RT-PCR with encephalomyocarditis virus as internal control. Total RNA/DNA was extracted from 200 µL of serum or plasma using the automated NUCLISENS easyMAG system (Biomérieux) and dissolved in 55 µL of purified water. For RNA amplification, 23.1 µL of RNA extract was used for real-time RT-PCR. A reverse transcriptase kit (Kit TaqMan Reverse Transcription Reagents, Applied Biosystems) was used to perform the reverse transcription of RNA to cDNA in a total volume of 60 µL. Reverse transcription profile: 10 min 25°C, 30 min 48°C, and 5 min 95°C. Of cDNA, 10 µL was used as a template in a final volume of 20 µL with Roche Probes Master (Roche Diagnostics Nederland BV, Almere, the Netherlands). The assay was performed with LightCycler480 (Roche diagnostics Nederland BV, Almere, the Netherlands) with the following real-time RT-PCR profile: 45 cycles of 95°C for 15 s and 60°C for 60 s. The threshold for positivity was calculated and set using the LightCycler480 software (Roche diagnostics Nederland BV, Almere, the Netherlands), and samples were considered positive if the threshold was reached within 40 amplification cycles [cycle threshold (Ct) ≤40] .

### Algorithms

Confirmed diagnoses either by real-time RT-PCR or follow-up serology were taken as endpoint for clinical means. Presumptive cases were defined as being tested IgM positive. Subsequently, we constructed and evaluated two potential algorithms incorporating orthohantavirus real-time RT-PCR in routine diagnostics, one that was supposed to yield the highest number of presumptive and confirmed diagnoses on the acute serum sample with minimal delay, by performing serology and real-time RT-PCR testing at the same time. The other algorithm was designed to reduce laboratory costs and efforts, using a two-step approach, starting with serology followed by real-time RT-PCR testing at a later stage. The likelihood of a positive real-time RT-PCR result in relation to the duration of symptoms was evaluated, and a conditional involving the time after onset of symptoms was integrated in the algorithms.

To demonstrate the benefits of these algorithms, a selection of sera, namely, those with a known PCR result (*n* = 85), was used for modeling and run through either algorithm. The results were compared with the current strategy that uses serology alone.

## RESULTS

### Detection of orthohantavirus RNA

Of 113 sera of clinically suspected hanta virus cases, 24 sera tested positive for anti-PUUV IgM antibodies, and 89 sera tested negative or indeterminate (Table 2). Of 16 of the IgM-positive sera that were available for real-time RT-PCR, eight sera tested positive with PUUV real-time RT-PCR (Tables 1 and 2). Ct values ranged from 36 to 40 cycles (data not shown). For seven out of eight real-time RT-PCR-positive cases, the time from onset of symptoms to collection of the first serum sample was 7 days or less. For one of eight real-time RT-PCR-positive cases, the time of onset of symptoms was unknown. In five out of eight anti-PUUV IgM-positive cases that tested negative with PUUV real-time RT-PCR, blood was collected within 7 days after onset of symptoms. In one case, the time of blood collection was more than 7 days after the onset of symptoms. In two cases the time of onset was unknown (Table 3).

**TABLE 2:** Real-time RT-PCR results for endemic orthohantavirus species on sera from orthohantavirus infection-suspected patients in the Netherlands

Sera tested for anti-PUUV IgM (*n* = 113)		Number of sera tested with real-time RT-PCR (%)	PUUV real-time RT-PCR positive sera (% of tested sera)	TULV real-time RT-PCR positive sera	SEOV real-time RT-PCR positive sera
Positive	Negative or indeterminant				
24		16 (67%)	8 (50%)	0	0
	89	69 (78%)	1 (1.4%)	0	0

**TABLE 3:** Time between onset of symptoms to blood collection of patients with anti-PUUV IgM antibodies in the first serum sample

Positive PUUV real-time RT-PCR		Negative PUUV real-time RT-PCR	
Serum number	Time from onset of symptoms to blood collection (days)	Serum number	Time from onset of symptoms to blood collection (days)
1	5	4	7
2	1	5	Unknown
3	3	6	1
16	<7	7	14
17	5	12	7
18	Unknown	13	5
19	7	21	4
20	<7	22	Unknown

Of 69 of the 89 sera that tested serologically negative or indeterminate for PUUV IgM antibodies, which were available for real-time RT-PCR, one serum tested positive with PUUV real-time RT-PCR (Table 2, serum 25 in Table 1). This serum was obtained 2 days after onset of symptoms (not shown).

In all PUUV real-time RT-PCR-positive cases with a known time of onset of disease (7/8), the first serum sample was taken on or before the 7th day of disease (Table 3).

None of the sera tested positive for SEOV or TULV with real-time RT-PCR (Table 2).

### Follow-up serology

In 10/113 cases, a follow-up sample was sent to the laboratory for evaluation (Table 1). Time intervals between the two serum samples ranged from 7 to 49 days. In 5/10 cases, follow-up serology confirmed PUUV infection, and in 5/10 cases, infection with PUUV could be excluded based on a negative follow-up sample (Table 1). In one of the serologically positive confirmed cases, the first serum sample could not be tested for PUUV RNA because of the limited amount of serum available (Serum number 27 in Table 1).

### Diagnostic algorithms

**Fig 1:**
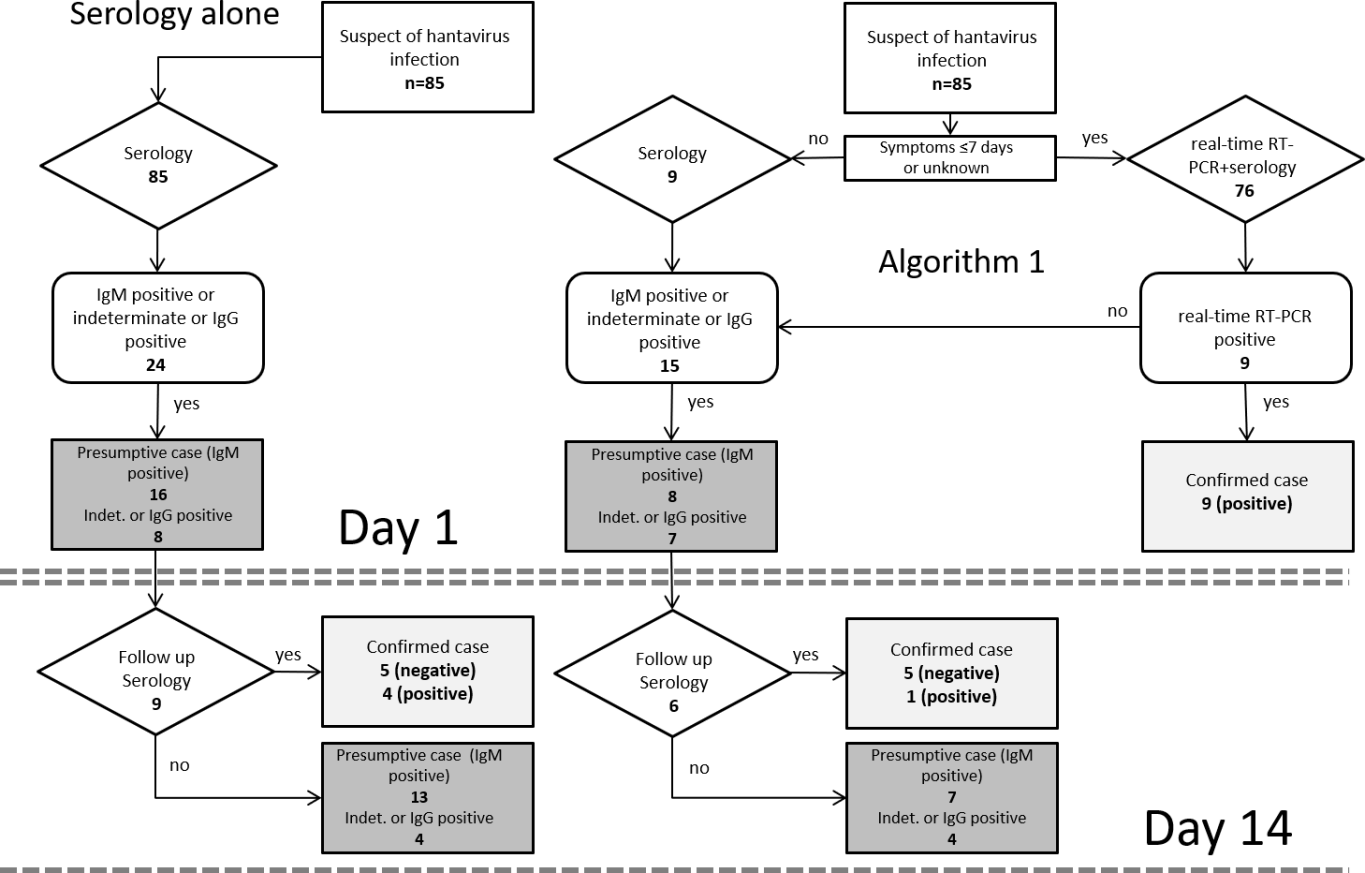
Algorithm 1: If suspected for orthohantavirus infection and if symptoms exist for 7 days or less, perform real-time RT-PCR and serology simultaneously. If real-time RT-PCR is negative, perform serology on a second sample after 2 weeks for a confirmed diagnosis. Data from real-time RT-PCR-tested sera from patients who had been routinely tested by serology previously (*n* = 85) were run through the algorithm as a model (on the right); the resulting numbers of confirmed cases and presumptive cases at a given time are depicted in the light gray boxes and dark gray boxes, respectively, and may be compared with those resulting from the strategy of serology alone (on the left). Indeterminate (indet.) or IgG-positive cases generally require further evaluation, like the presumptive cases, and are depicted in the same dark gray box.

**Fig 2:**
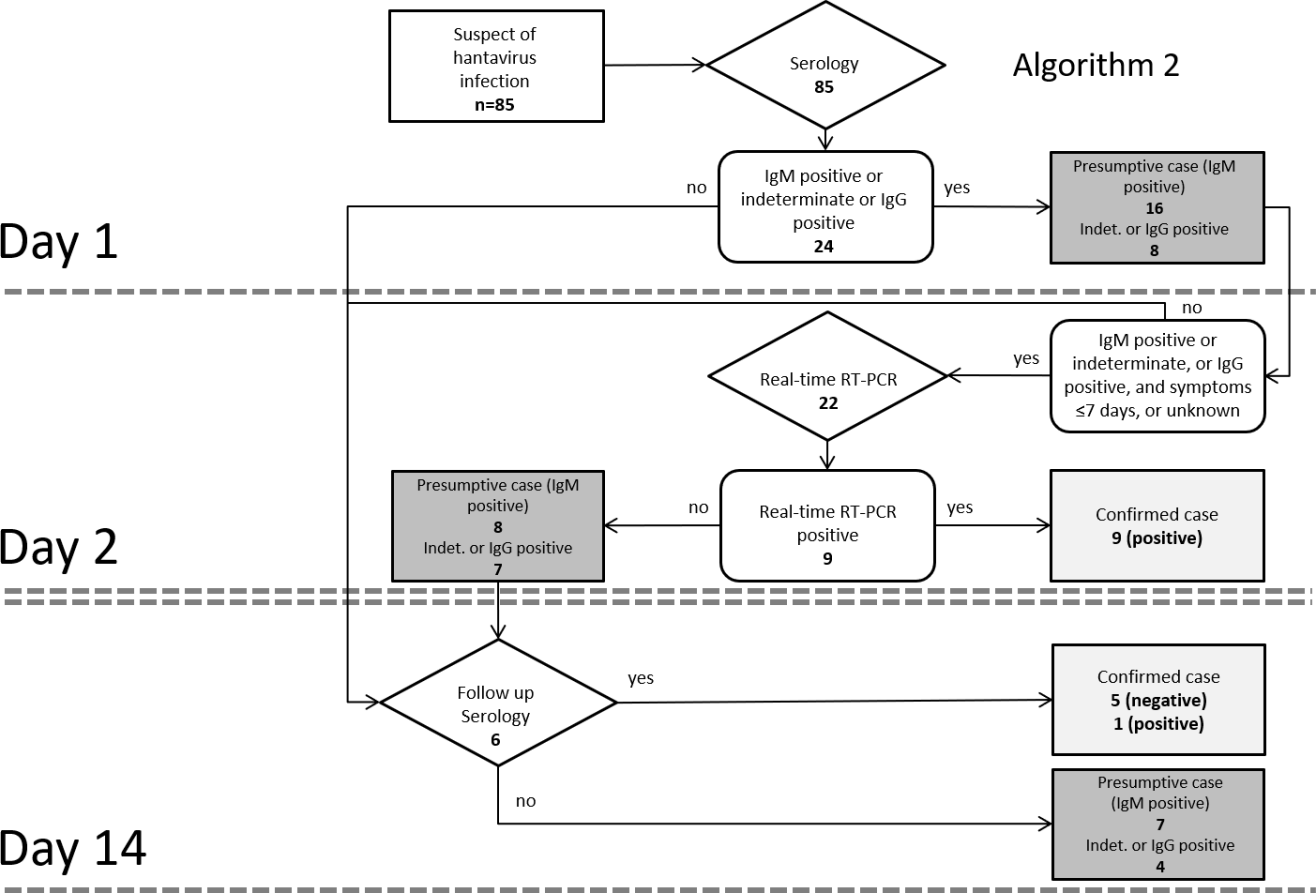
Algorithm 2: If suspected for orthohantavirus infection, perform serology on day 1. Perform real-time RT-PCR on day 2 on the IgM-positive and indeterminate sera of patients who have symptoms for 7 days or less. If real-time RT-PCR is negative, perform serology on a second sample after 2 weeks for a confirmed diagnosis. Data from real-time RT-PCR-tested sera from patients who had been routinely tested by serology previously were run through the algorithm as a model; for explanation of the given numbers of cases and abbreviations, see Fig. 1.

Algorithm 1 (Fig. 1) was designed to yield the maximum number of positive samples (presumptive and confirmed cases) on the first day of testing, by submitting all samples to real-time RT-PCR as well as serology testing at the same time. Because all samples that tested positive with real-time RT-PCR had been taken 7 days or less after onset of symptoms, a conditional was introduced in the algorithm to prevent apparently unnecessary testing after 7 days post-onset. PUUV infections missed by real-time RT-PCR on day 1 were expected to be confirmed by follow-up serology, supposedly on day 14. In Algorithm 2 (Fig. 2), all samples were tested by serology on the first day, and all serologically negative samples were excluded from real-time RT-PCR testing further on because none of the serologically negative tested samples were found positive using real-time RT-PCR testing. Presumptive cases and indeterminate or IgG-positive cases were tested subsequently by real-time RT-PCR, likely to be performed on the next day. Like in algorithm 1, serology on a follow-up sample after presumably 14 days was included to confirm possible PUUV infections missed by real-time RT-PCR.

All 85 sera with a known PUUV real-time RT-PCR result (positive or negative) were run through both algorithms 1 and 2 to compare the differences in timing and number of confirmed and presumptive diagnoses to be revealed by both algorithms, and this was compared to the strategy of serology alone (Fig. 1 and 2). Information on the time after onset was available only for 41 of the 85 sera [IgM and IgG negative (*n* = 61): 18 (≤7 days) and 7 (≥7 days); IgM-positive sera (*n* = 16): 12 (≤7 days) and 1 (≥7 days; see also Table 3); IgM indeterminate or IgG-positive sera (*n* = 8): 2 (≤7 days) and 1 (≥7 days)]. For practical reasons, the sera for which the time after onset was not known were treated as if ≤7 days after onset. Numbers of indeterminate or IgG-positive samples, which do not indicate a presumptive case, but would require further evaluation, were presented in the same box as the presumptive cases. On day 1 of testing according to algorithm 1, nine confirmed cases of PUUV infection were revealed, with eight presumptive cases and seven indeterminate or IgG-positive cases, compared to standard serology alone, which showed no confirmed cases on day 1 but 16 presumptive cases and eight indeterminate or IgG-positive cases. In algorithm 1, follow-up serology revealed another confirmed positive case and five confirmed negative cases after 14 days. Using standard serology alone, a total of four confirmed positive cases were revealed after 14 days, compared to 10 confirmed positive cases using algorithm 1. Using algorithm 2, the same total number of confirmed positive samples (*n* = 10) was revealed as was using algorithm 1 (Fig. 1). However, the majority (*n* = 9) of confirmed positive cases was revealed with 1 day delay (on day 2 vs day 1). In algorithm 2, only 22 sera were tested with real-time RT-PCR, compared to 76 sera tested in algorithm 1, which represents a 71% reduction in real-time RT-PCR tests performed.

Follow-up serology was only available for 9/85 real-time RT-PCR tested samples (see also the Materials and Methods section) revealing five confirmed negative cases and four confirmed positive cases (Fig. 1, serology alone). In algorithm 1 and 2, three of these four serologically confirmed positive cases were already identified in an earlier stage by real-time RT-PCR.

The time of confirmation of PUUV infection and the comparative numbers of confirmed positive cases in this model expressed as percentages of the maximum of confirmed positive cases (*n* = 10) are summarized in Table 4.

**TABLE 4:** Efficacy of the combination of real-time RT-PCR detection and serology in revealing confirmed positive cases of acute orthohantavirus infection

Test combination		Percentage of maximum number of confirmed cases (real-time RT-PCR-positive and/or confirmed by serology) at given day of testing		
		Day 1	Day 2	Day 14
Serology alone				40% (4/10)
Serology and real-time RT-PCR	Algorithm 1	90% (9/10)		100% (10/10)
	Algorithm 2		90% (9/10)	100% (10/10)

## DISCUSSION

A potential role for real-time RT-PCR in orthohantavirus diagnostics in the regions of *Twente* and *Achterhoek* in the Netherlands was evaluated using stored serum samples, which have been screened previously for orthohantavirus infection with routine serological assays (ELISA or IF). In 50% of anti-PUUV IgM-positive serum samples, PUUV RNA was detected by real-time RT-PCR, in retrospect, confirming an acute infection at that time. All real-time RT-PCR-positive sera, for which a specified disease history was known, had been collected early in the disease course, i.e., within 7 days after onset of symptoms. Other studies show that the majority of PUUV RNA-positive serum samples may be detected within the first 7 to 8 days after symptomatic onset, although, occasionally, PUUV RNA has been detected up to 16 days after onset of symptoms (17–20). The sensitivity of PUUV NAAT varies in literature and may be influenced by differences in test conditions, including the timing of sample collection, but also by genetic differences in PUUV strains circulating in different endemic areas or the level of viremia induced by certain strains (17–20). In a Swedish study, the same real-time RT-PCR that detected PUUV RNA in 97% (33/34) of PUUV IgM-positive (IF) serum samples of NE patients in the north of Sweden only did so in 40% (4/10) of the cases in the south of Sweden, where a distinct genetic lineage of PUUV circulates (19). Similarly, using primers designed for Swedish PUUV strains, PUUV RNA was only detected in 12.5% (1/8) of serologically positive Norwegian serum samples (21).

Optimizing the PUUV real-time RT-PCR to increase its sensitivity for PUUV strains within a certain region may be worthwhile. Recently, Lagerkvist et al. designed a real-time RT-PCR using all published PUUV S segment sequences of Swedish origin, which detected PUUV RNA with 98.7% sensitivity and 100% specificity in serum samples from patients from all over Sweden taken within 8 days after onset of HFRS (17). Similarly, Niskanen et al. evaluated a one-step real-time RT-PCR (PUUV-qRT-PCR), adjusted to strains circulating in Finland, and found a sensitivity of 93.3% and specificity of 100% (18). We used a real-time RT-PCR designed by Kramski et al. (16), and it may be that primers and probes are suboptimal to detect the PUUV strain(s) in our region. However, we were able to detect RNA from PUUV derived from two mice caught in the *Twente* region (data not shown) with current real-time RT-PCR settings. Still, it would be interesting to see if the performance of the real-time RT-PCR may be improved by adjusting the primers and probes to PUUV strains circulating in specific regions in the Netherlands, as for instance in the *Twente* region. Furthermore, primers and probes optimized to detect strains from different regions could possibly be combined in a multiplex real-time RT-PCR test to create a test that is better suited for larger parts of the country. On the other hand, freeze-thawing effects on the viral RNA in the serum samples may have contributed to a decreased sensitivity of the real-time RT-PCR in our study. Whether application of the real-time RT-PCR on fresh serum samples might yield a higher percentage of positives needs to be proven.

Serology may be false positive, as has been shown for another hantavirus IgG ELISA test (5), and the true number of acute orthohantavirus infections may be lower than the number of IgM-positive sera would suggest. Unfortunately, in only three of the 24 PUUV IgM-positive cases, a follow-up sample was provided for confirmation. In one of these three cases, an acute infection could not be confirmed either by serology on a follow-up sample or using real-time RT-PCR testing. This shows that relying on a positive IgM test on the first serum sample alone can lead to an overestimation of the number of acute cases. Consequently, with the current real-time RT-PCR test, the proportion of true acute PUUV infections identified by real-time RT-PCR among the IgM-positive cases is probably greater than 50%.

For clinical practice, the current real-time RT-PCR may not be sensitive enough to be used as a single test in routine orthohantavirus diagnostics, while serology alone cannot confirm the diagnosis in the early phase of the disease, and sera may be false positive for anti-PUUV IgM. However, a combination of both tests could provide the clinician with more optimal information for proper patient care, as was exemplified by a model using the two suggested algorithms. Combining serological screening with real-time RT-PCR on the same day in routine diagnostics yielded the maximum numbers of confirmed orthohantavirus infections on a single serum sample on the first day of testing (Algorithm 1, see Fig. 1). With this algorithm, also cases with a false-negative or indeterminate anti-PUUV IgM but with a positive PUUV real-time RT-PCR result would be revealed on the first day of testing, as was shown to be the case for one indeterminate serum sample (1.4%; 1/69) in our study. However, performing both serology and real-time RT-PCR on every sample in routine diagnostics will increase testing efforts and costs significantly, compared to serology alone. Costs of a real-time RT-PCR test, in general, may be a multiple of the costs of standard serologic tests (IgG and IgM combined). To economize, this algorithm real-time RT-PCR testing was restricted to samples taken within 7 days after onset of symptoms. However, in the current model, this conditional reduced the amount of real-time RT-PCR testing with only 11% (9/85 samples).

To reduce the efforts and costs of testing more significantly, sera could first be routinely screened with serologic tests, and real-time RT-PCR could then be performed only on PUUV IgM and/or IgG-positive or indeterminate sera, to confirm the diagnosis (Algorithm 2, see Fig. 2). In this way, in the current model, 22 sera were tested using real-time RT-PCR compared to 76 sera when algorithm 1 was used. The use of algorithm 2 could therefore result in a 71% reduction in efforts and costs compared to that of algorithm 1. Downsides of this strategy include a possible delay, most likely of a day, and missing of sporadic cases with a false-negative IgM but positive real-time RT-PCR, which have been found by others (17). Independent of the algorithm used, implementing real-time RT-PCR in routine orthohantavirus diagnostics would reveal the majority of confirmed orthohantavirus infections on the first serum sample and overall 60% more confirmed diagnoses than can be expected using serology alone in the current general practice. Combining orthohantavirus serology with real-time RT-PCR therefore maximizes the impact of timely laboratory testing and may help avoid invasive procedures in patients presenting with severe acute kidney failure.

Aside from in blood samples, PUUV RNA has been detected using real-time RT-PCR in excreta of infected patients like urine and saliva. Although urine was found inadequate for routine diagnostics in a recent study by others (22), a role for saliva still needs to be determined. Interestingly, during an outbreak in northern Sweden, 10 of 14 patients hospitalized with NE who had PUUV RNA-positive blood samples also had their saliva tested positive for PUUV RNA (23).

None of the sera tested positive for TULV or SEOV viral RNA using real-time RT-PCR, which does not rule out infection with these virus types. For instance, strain differences between orthohantaviruses used to develop the real-time RT-PCR and circulating strains in the *Twente* and the *Achterhoek* regions may influence the real-time RT-PCR sensitivity, similar to the PUUV real-time RT-PCR in the study by Lagerqvist et al. (17). SEOV detected in wild rats in the *Achterhoek* region belonged to lineage #9, while the primers and probes used in this study had been developed using SEOV of lineage #4 (8, 16, 24). On the other hand, clinical presentation of TULV and SEOV may differ substantially from PUUV infection, questioning if the currently selected sera represent the optimal sera to detect TULV and SEOV cases (15).

For the detection of possible SEOV infections, the time window to detect SEOV RNA in the blood is considered to be wider than for PUUV, further decreasing the chance of a missed diagnosis (15). To further exclude any missed SEOV cases in this selection of sera, additional serological test for anti-SEOV antibodies should be performed. Yet, the risk of contracting an SEOV infection from wild animals in the Netherlands is considered low; in a study among Dutch muskrat and coypu trappers in 2016 to estimate the health risks, no serologic evidence was found of exposure to SEOV, possibly due to the low prevalence of SEOV (10). The optimal time window to detect TULV RNA in serum is unknown. Only four human cases of TULV infection have been reported in literature (25–28). TULV viral RNA was detected in the blood 4 days and 11 days after onset of symptoms in immunocompetent and immunocompromised patients, respectively (27, 28).

In conclusion, implementation of real-time RT-PCR in routine orthohantavirus diagnostics is expected to confirm the diagnosis of acute orthohantavirus infection in about half of the serologically suspected cases, in the acute phase of the disease, when the impact on clinical decision making is highest. This may help prevent unnecessary invasive diagnostic and therapeutic procedures and reduce healthcare costs. Optimizing the real-time RT-PCR’s primers and probes to the locally circulating PUUV strains may further increase the benefit of real-time RT-PCR in routine diagnostics.
